# Growth factor choice is critical for successful functionalization of nanoparticles

**DOI:** 10.3389/fnins.2015.00305

**Published:** 2015-09-02

**Authors:** Josephine Pinkernelle, Vittoria Raffa, Maria P. Calatayud, Gerado F. Goya, Cristina Riggio, Gerburg Keilhoff

**Affiliations:** ^1^Department of Nephrology and Hypertension, Diabetes and Endocrinology, Otto-von-Guericke University of MagdeburgMagdeburg, Germany; ^2^Institute for Biochemistry and Cell Biology, Otto-von-Guericke University of MagdeburgMagdeburg, Germany; ^3^Department of Biology, University of PisaPisa, Italy; ^4^Institute of Life Science, Scuola Superiore Sant' AnnaPisa, Italy; ^5^Aragon Institute of Nanosciences, University of ZaragozaZaragoza, Spain; ^6^Department of Condensed Matter Physics, University of ZaragozaSpain

**Keywords:** functionalization, nanoparticles, glial cell-line derived neurotrophic factor (GDNF), nerve growth factor (NGF), organotypic spinal cord culture, PC12 cells

## Abstract

Nanoparticles (NPs) show new characteristics compared to the corresponding bulk material. These nanoscale properties make them interesting for various applications in biomedicine and life sciences. One field of application is the use of magnetic NPs to support regeneration in the nervous system. Drug delivery requires a functionalization of NPs with bio-functional molecules. In our study, we functionalized self-made PEI-coated iron oxide NPs with nerve growth factor (NGF) and glial cell-line derived neurotrophic factor (GDNF). Next, we tested the bio-functionality of NGF in a rat pheochromocytoma cell line (PC12) and the bio-functionality of GDNF in an organotypic spinal cord culture. Covalent binding of NGF to PEI-NPs impaired bio-functionality of NGF, but non-covalent approach differentiated PC12 cells reliably. Non-covalent binding of GDNF showed a satisfying bio-functionality of GDNF:PEI-NPs, but turned out to be unstable in conjugation to the PEI-NPs. Taken together, our study showed the importance of assessing bio-functionality and binding stability of functionalized growth factors using proper biological models. It also shows that successful functionalization of magnetic NPs with growth factors is dependent on the used binding chemistry and that it is hardly predictable. For use as therapeutics, functionalization strategies have to be reproducible and future studies are needed.

## Introduction

Nanoparticles (NPs) are materials that display different characteristics at the nanoscale than the corresponding bulk material. Usually, the term NP is used for substances with sizes about 1–100 nm, but there is an ongoing discussion about using the term NPs in a more size-independent way, referring more to the occurrence of new properties or the high ratio of surface area to volume (Kreyling et al., 2010). One feature that changes with nanoscale is magnetism. Iron is a ferromagnetic bulk material, however reducing its size to the nanoscale results in superparamagnetic behavior. Superparamagnetic NPs are non-magnetic until they are exposed to a strong magnetic field. If the magnetic field is removed, they revert to a non-magnetic state again (Mortimer and Müller, 2003; Estelrich et al., 2015). In the case of magnetite Fe_3_O_4_ and maghemite γ-Fe_2_O_3_, which are commonly used iron oxide NPs, the superparamagnetic diameter is 25 nm (Fe_3_O_4_) and 30 nm (γ-Fe_2_O_3_). Both iron oxides are highly used in biomedicine due to their stability, relatively low toxicity, and high magnetization.

Superparamagnetic iron oxide NPs can be used in diagnostic magnetic resonance imaging (MRI), e.g., Resovist® underwent successfully clinical trials and is used to enhance the contrast in MRI scans of the liver (Reimer and Balzer, 2003; Weinstein et al., 2010). In addition to tissue imaging, magnetic NPs are also used to track cells with the MRI *in vivo* (Dunning, 2004; Antonelli et al., 2013).

In cancer treatment, superparamagnetic NPs are used to induce hyperthermia in prostate cancer and glioblastoma (Jordan et al., 2001; Thiesen and Jordan, 2008). NPs are injected directly into tumor tissue and stimulated with an alternating magnetic field. This causes the particles to heat up, thereby destroying the tumor tissue.

To localize functionalized magnetic NPs within a tissue of interest, a static magnetic field can be applied. Chertok et al. (2008) followed intravenously injected NPs in rats with glioblastoma via MRI and showed an accumulation of NPs in the tumors. Cells can also be targeted magnetically. Hamasaki et al. (2007) labeled neural progenitor cells with magnetic NPs and localized them in an organotypic co-culture with the help of an external magnetic field. Corticospinal axon growth was improved by magnetically targeted neural progenitor cells in comparison to non-labeled cells. Labeling cells with magnetic NPs can also help to purify primary cell cultures. Gordon et al. (2011) used magnetic NPs to purify microglia from mixed primary glial cultures.

The magnetofection technique uses magnetic force to deliver nucleic acids or viruses into cells. By applying a strong external magnetic field, cells can be transfected with superparamagnetic NPs, which are functionalized with gene vectors (Scherer et al., 2002). Magnetofection offers a simple method to transfect cells that are normally difficult to transfect, e.g., cells of the central nervous system (CNS). Efficient transfection was achieved in primary neural stem cells (Sapet et al., 2011), oligodendrocyte precursor cells (Jenkins et al., 2011), hippocampal neurons (Buerli et al., 2007), and astrocytes (Pickard and Chari, 2010). Also primary motor neurons could be transfected using this method. Fallini et al. (2010) transfected primary motor neurons with NPs functionalized with GFP-expressing plasmids. Motor neurons showed no signs of cytotoxicity and about 45% of cells could be transfected.

Magnetic NPs have a large surface-to-volume ratio that enables chemical conjugation and changes the surface properties of the NPs. Usually; they are synthesized by wet chemistry approaches, which produce Ferro fluid water dispersions (Vergés et al., 2008). The stability of Ferro fluids depends on the equilibrium between dipole-dipole interactions among particles and particle-solvent interactions. In order to decrease the strength of dipole-dipole interactions and stabilize the water dispersion of NPs as single particles or small clusters, a surface coating is required. The coating increases the hydrodynamic ratio of the particles, which decreases magnetic interactions among particles, stabilizing the dispersion. Additionally, surface properties affect biocompatibility, particle opsonization in biological media (Tenzer et al., 2013), cellular internalization mechanisms, and biological interactions (Gao et al., 2009; Veiseh et al., 2010). For coating, organic polymers (dextran, chitosan, polyethylene glycol), inorganic substances (gold, silica, carbon), and bioactive molecules (liposomes, proteins, ligands) can be used (Shubayev et al., 2009; Estelrich et al., 2015). The functionalization with bioactive groups and proteins allows a wide range of applications, particularly in life sciences (Pankhurst et al., 2003; Gupta and Gupta, 2005). Drug delivery is one application of interest (Arruebo et al., 2007; Estelrich et al., 2015). Huang et al. (2015a) described layer-by-layer casein-coated magnetic NPs, which could be loaded with doxorubicin and indocyanine green. NPs were stable under gastric conditions and drugs were released by degradation of the casein in the intestine. Nazli et al. (2014) showed that doxorubicin-delivery by MMP-sensitive PEG hydrogel-coated magnetic NPs are taken up efficiently into HeLa cells and the drug released within 2 h.

In neurosciences, magnetic NPs have received attention in the fields of CNS and peripheral nervous system (PNS) regeneration. Injury to the nervous system produces high costs for the health systems, partially due to massive lifelong impairments (Noble et al., 1998; Wyndaele and Wyndaele, 2006) and therefore is still a highly interesting field of investigation.

In CNS injury, one distinguishes between primary and secondary injury. The primary injury results from mechanical forces, damaging cells at the injury site. Due to secondary processes the size of the injury is increased and damage is prolonged. Tissue is additionally damaged by ischemia, edema, excitotoxicity, shifting of ion concentrations, production of reactive oxygen species, inflammation, necrosis, and demyelination of axons (Salewski et al., 2013; Siddique and Thakor, 2014).

Regeneration in the CNS is a huge challenge. One reason for this is the complex glial composition of the CNS: astroglia, microglia, and oligodendroglia. The inhibition of oligodendroglia and its apoptosis after loss of axon contact (Beattie et al., 2000; Vargas and Barres, 2007), the activation of microglia, which secrete pro-inflammatory cytokines (Danton and Dietrich, 2003), and the activation of astroglia, which results in the formation of a glia scarring in the injured area, are producing a regeneration-inhibiting environment making regeneration impossible (Fawcett and Asher, 1999; Fitch and Silver, 2008). In addition to surgical therapies, therapy approaches often try to modulate inflammatory actions, e.g., using minocycline or methylprednisolone (McDonald and Sadowsky, 2002; Beattie, 2004; Stahel et al., 2012). Studies trying to influence the glial scar and the regeneration-inhibiting features of residual myelin are done, too (Kwon et al., 2011). Nevertheless, neurons of the CNS have the capacity to regenerate, which was demonstrated by mixing CNS and PNS tissue/cells. In a mixed milieu, CNS neurons can regenerate into a PNS environment (David and Aguayo, 1981; Pearse et al., 2004).

In contrast, the potential for regeneration in the peripheral nervous system is, in principle, good (Navarro et al., 2007; Vargas and Barres, 2007; Svennigsen and Dahlin, 2013). After axon injury in the peripheral nerve several processes of degeneration and regeneration start. The injured neuron undergoes changes in metabolism and protein expression after retrograde signaling and calcium influx at the lesioned axon. Chromatolysis occurs and regeneration-related proteins like growth-associated protein 43 are upregulated. Growth cones are formed and elongate following guidance molecules provided by activated Schwann cells (Dent et al., 2003; Bradke et al., 2012; Patodia and Raivich, 2012). Schwann cells are activated by losing axonal contact and interruption of blood and oxygen supply. Calcium signaling activates intracellular cascades including mitogen-activated protein kinases (MAPK), like the extracellular signal-regulated protein kinases (ERK1/2), and c-jun N-terminal protein kinases (JNK1/2/3) (Agthong et al., 2006; Chattopadhyay and Shubayev, 2009; Yamazaki et al., 2009; Arthur-Farraj et al., 2012). These signaling cascades result in a termination of myelin production, dedifferentiation, and then proliferation of Schwann cells at the distal injury site. Schwann cells start phagocytosis of myelin and cell debris before macrophages enter the injury site time-delayed. Schwann cells then start to produce regeneration-promoting factors, like nerve growth factor (NGF), brain-derived growth factor (BDNF), and glial cell-line derived neurotrophic factor (GDNF) (Funakoshi et al., 1993; Xu et al., 2013; Huang et al., 2015b), and align to regular structures, e.g., the Bands of Büngner, which guide regenerating axons to reinnervate their targets. Altogether, Schwann cells help to establish a regeneration-permissive environment for the regenerating axon.

Nevertheless, the regenerative outcome in the PNS is poor. Only 40% of patients regain a normal functionality, 16% show a good functional outcome, whereas 32% of the patients show little improvement, and 12% even show a complete loss of function (Noble et al., 1998). The clinical strategies used to treat patients with peripheral nerve injury have not improved in the last years. Surgery is still the treatment of choice (Scholz et al., 2009). Direct end-to-end repair avoiding tensions shows the best outcome. If tensionless repair is not possible, grafting or conduits are chosen to bridge the gap between the nerve endings. Often sensory nerves from the patient (autograft) or from donors (allograft) are used as grafts. Blood vessels are usually used as biological conduits. Collagen, polyglycolic acid, and caprolactone conduits are also available (Dahlin, 2008; Griffin et al., 2013; Grinsell and Keating, 2014).

One problem, even after successful surgery, is the non-specific reinnervation of target organs due to randomly axonal sprouting and misdirection of regenerating axons. Additionally, projections in the CNS are lost after an injury and new synaptic contacts need a rebuilding of CNS projections and a long-term learning process (Navarro et al., 2007; de Ruiter et al., 2014).

Magnetic NPs now offer a new possibility to promote PNS regeneration. One idea is to facilitate the uptake of magnetic NPs in regenerating neurons and their axons and to pull the axon along an external magnetic field. Therefore, the guidance of sprouting axons is promoted and the specificity of reinnervation is enhanced (Halpern, 2000; Riggio et al., 2012; Calatayud et al., 2013; Goya et al., 2014; Riggio et al., 2014). Previous studies showed initial success. Human neuroblastoma (SH-SY5Y) cells were effectively influenced by an external magnetic field after magnetic NPs uptake. Also primary Schwann cells and olfactory ensheating cells showed satisfying uptake of NPs and could be moved along a magnetic field (Riggio et al., 2012, 2013). Neurite orientation was shown to be directed toward a magnetic force after uptake of magnetic NPs into differentiated PC12 cells (Riggio et al., 2014).

However, peripheral nerve injury is more complicated. To facilitate regeneration of injured axons, NPs have to be taken up into neurons. Primary neuronal cells in mixed cell cultures showed relatively low uptake of iron oxide NPs (Pinkernelle et al., 2012). Petters and Dringen (2015) showed that using serum-containing media significantly lowers the uptake of magnetic iron oxide NPs in cerebellar granule neurons compared to serum-free media. NPs diluted in serum-containing media develop a protein corona, which seems to impede the efficient uptake of NPs in neurons. There are various strategies to enhance neuronal uptake. Adams et al. (2015) increased magnetite content of NPs using sedimenting forces. Buerli et al. (2007) and Fallini et al. (2010) used magnetofection to promote uptake into neurons and transfected them with DNA. Another possibility is the coupling of growth factors, which utilize receptor-mediated uptake. Ziv-Polat et al. (2014) conjugated iron oxide NPs with different neurotrophic factors. They showed that the stability of neurotrophic factors in media and their functional activity was improved by binding them to the NPs. Altogether coupling of growth factors seems to be the most promising strategy in terms of a future clinical approach.

In our study, we focus on 2 growth factors: NGF and GDNF. Both play a role in PNS regeneration. NGF belongs to the neurotrophin family binding to the tropomyosin receptor kinase A (TrkA) or to p75 receptor (Boyd and Gordon, 2003). Most studies concerning NGF were performed in PC12 cells, which express both TrkA and p75 receptors. They can be differentiated into neuronal-like cells displaying neurite development (Greene and Tischler, 1976; Dichter et al., 1977). Signaling of Trk receptors is mediated via the activation of phosphatidylinositol 3-kinase (PI3K), MAPK, and phospholipase Cγ (PLCγ). These pathways regulate differentiation, survival, and neuritogenesis (Boyd and Gordon, 2003).

GDNF is a neurotrophic factor belonging to the GDNF family. These growth factors are secretory proteins and bind to a receptor complex consisting of a high affinity ligand-binding subunit, the GDNF family receptor-α (GFR-α), and a signal transduction subunit RET receptor tyrosine kinase. GDNF binds to GFR-α1 thereby activating RET, which leads to the activation of the intracellular tyrosine kinase domain. PI3K, MAPK, and PLCγ pathways are activated, regulating cell survival, neurite outgrowth, and synaptic plasticity among other activities (Airaksinen and Saarma, 2002; Boyd and Gordon, 2003; Sariola and Saarma, 2003).

We used self-made iron oxide NPs coated with polyethyleneimine (PEI) for coupling growth factors (Calatayud et al., 2013). PEI is a polymer, which is also used for the transfection of cells (Vancha et al., 2004). It contains several amine groups allowing covalent binding of proteins via peptide bonds. First, we choose NGF for functionalization. NGF can be internalized with its receptor (Neet and Campenot, 2001; Matusica and Coulson, 2014) and therefore, coupling of NGF to PEI-NPs should result in receptor-mediated uptake, increasing the uptake rate of the NPs.

To check for bio-functionality of NGF after coupling to the NPs, we used PC12 cells and induced the differentiation with the help of our functionalized PEI-NPs. However, these cells cannot be used to model the complex processes of neuronal injury and regeneration. Because of this, we also used a neonatal organotypic spinal cord model to resemble the regeneration of motor neurons after axotomy (Pinkernelle et al., 2012, 2013; Keilhoff et al., 2014). Organotypic cultures keep the cellular organization and the cell-cell-contacts. Therefore, they model the *in vivo* environment more accurately than disperse primary cell cultures or cell lines (Stavridis et al., 2005; Pinkernelle et al., 2013). Proximal axotomy was induced through the preparation of the spinal cord slices and resulted in a loss of motor neurons. The trophic requirements of motor neurons are still under discussion due to the variety of factors needed and the wide range of cells providing them (Oppenheim, 1996; Brunet et al., 2007).

GDNF is one of the most prominent neurotrophic factors playing a role in both the survival of motor neurons and their axonal regeneration (Bohn, 2004; Brunet et al., 2007; Vyas et al., 2010; Pajenda et al., 2014). In organotypic spinal cord cultures, a representative motor neuronal population of about 60% can be found after 1 week of cultivation with GDNF supplement (Rakowicz et al., 2002; Vyas et al., 2010). Therefore, we functionalized our self-made PEI-NPs with GDNF. Like NGF, GDNF may be internalized via endocytosis together with its receptor and moves anterogradely along axons (Neet and Campenot, 2001; von Bartheld et al., 2001). In previous studies, we found no reliable uptake of iron oxide NPs in primary motor neurons and granular cells (Pinkernelle et al., 2012). Therefore, we tried to facilitate the uptake of magnetic PEI-NPs with the help of GDNF functionalization in order to obtain a receptor-mediated uptake of NPs into the motor neurons of our spinal cord model.

## Materials and methods

### Cell cultures

#### Cultivation

PC12 cells obtained from American Type Culture Collection were cultured in Dulbecco's modified Eagle's media with 10% horse serum, 5% fetal bovine serum (FBS), 100 IU/ml penicillin, 100 μg/ml streptomycin, and 2 mM L-glutamine. Cells were cultivated in poly-L-lysine (Sigma, St. Louis, USA) coated dishes and maintained at 37°C in a saturated humidity atmosphere of 95% air and 5% CO_2_. For cell differentiation, PC12 cells were incubated in serum-reduced media (2% FBS).

#### PC12 actin staining

The cytoskeletal arrangement of PC12 cells was studied by means of actin staining. PC12 were seeded in Petri-dishes (for confocal microscopy) at a concentration of 5 × 10^5^ cells/ml and incubated overnight in growth media for cell adhesion. NGF:PEI-NPs were added in reduced serum media at a concentration of 10 μg/ml. Cells were cultured for 3 days before staining.

The media was removed and the cells gently washed with phosphate buffered saline (PBS) at 4°C followed by fixation/permeabilization with a solution of methanol: acetone (1:1 v/v) at −20°C for 30 min. At the end of incubation, the cells were washed 3x with PBS at 4°C and incubated overnight at 4°C with the primary anti-actin antibody (1:200, rabbit polyclonal, Santa Cruz Biotechnologies, Santa Cruz, USA). Then, the sample was washed again 3x with PBS at 4°C and incubated 1 h at room temperature with the secondary antibody (1:100, goat anti-rabbit IgG-TR, Santa Cruz Biotechnologies). Cells were washed 3x with PBS, dried, and mounted. The images were analyzed by confocal microscopy.

#### Electron microscopy

Electron imaging was performed with a scanning electron microscope (SEM INSPECT F50.FEI company) and dual-beam (FIB/SEM. Helios 600.FEI company). SEM images were taken at 5 and 30 kV with a FEG column and a combined Ga-based 30 kV (10 pA) ion beam was used to cross-section single cells. The investigations were completed by energy-dispersive X-ray spectroscopy (EDX) for chemical analysis. For this, PC12 cells were seeded on glass coverslips (previously coated with poly-L-lysine) at a density of 5 × 10^5^ cells/ml. After cell adhesion, the growth media was removed and replaced with the reduced media containing the functionalized NPs (10 μg/ml) or media with corresponding NGF concentration. After 72 h of incubation, cells were washed with PBS, fixed, dehydrated, dried, and sputtered with 30 nm of gold for electron imaging.

### Organotypic spinal cord cultures and co-cultures

#### Animals

All animal studies were performed in accordance with the guidelines of the German Animal Welfare Act. This study was approved by the Animal Care and Use Committee of Saxony-Anhalt, Germany. A formal approval to conduct the described experiments was obtained from the Animal Subjects Review Board of our institution and can be provided upon request. All efforts were made to minimize the number of animals used and their suffering.

#### Preparation and cultivation

Organotypic cultures were prepared as described by Vyas et al. (2010) with slight modifications. Neonatal rats (postnatal day 4) were decapitated, and their spinal cords excised. Spinal roots and meninges were removed in dissection buffer (Hank's balanced salt solution, 3.4 mM NaHCO_3_, 10 mM 4-(2-hydroxyethyl)-1-piperazineethanesulfonic acid, 33.3 mM D-glucose, 5.8 mM MgSO_4_, 0.03% bovine serum albumin (BSA), 1% penicillin/streptomycin) and the lumbar spinal cord (approximately L1-L6) was cut into 350 μm transverse sections using a McIlwain tissue chopper (Mickle Laboratory Engineering, Gomshall, UK). 6–8 slices were used from 1 animal and distributed equal to all experimental groups. The slices were cultured on Millicell membrane inserts (Millipore, Billerica, USA) in 6-well plates. Each well contained 1 ml of media composed of 50% Eagle's minimal essential media, 25% Hank's balanced salt solution, 25% FBS, 35 mM D-glucose, 2 mM L-alanyl-L-glutamine, 1% penicillin/streptomycin. Cultures maintained at 37°C in a saturated humidity atmosphere of 95% air and 5% CO_2_.

Culture preparation induces a loss of motor neurons due to the proximal axotomy caused by the slicing. However, a representative population of motor neurons (approximately 60%) can be found after 1 week in culture in the presence of GDNF (R&D systems, Minneapolis, USA) (Rakowicz et al., 2002; Vyas et al., 2010). Thus, control cultures received GDNF treatment to stabilize the motor neuron population.

#### Immunohistochemistry

Cultures were fixed after 1 week of cultivation by replacing the media with 4% paraformaldehyde overnight. The membranes of the Millicell inserts were separated from the carrier and the cultures were stained free-floating. Slices were washed 3x with PBS. Non-specific binding sites were blocked with 10% FBS and 0.3% Triton-X100 in PBS for 1 h. Cultures were incubated overnight with primary anti-pan-neurofilament antibody (1:1000, mouse monoclonal, Sternberger Monoclonals, Baltimore, USA) to visualize neurons including motor neurons and neurites diluted in 10% FBS, 0.3% Triton-X100 in PBS. Next, 3x washing with PBS was followed by secondary antibody incubation for 3 h (1:250, goat anti-mouse Alexa 488, Invitrogen, Carlsbad, USA). The slices were washed again and embedded on glass slides with Immu-Mount (Thermo Scientific, Waltham, USA).

Cultures were imaged with an AxioImager microscope and analyzed with the AxioVision Rel. 4.8 Imaging software by Zeiss (Jena, Germany). The microscopic settings and the exposure time were set on the basis of control slices and kept equal for the corresponding preparation.

Statistical analysis was performed using Graph Pad Prism 4 (GraphPad Software, La Jolla, USA).

### Nanoparticles

#### Synthesis of PEI-NPs

Magnetic NPs synthesis is based on a modification of the well-established oxidative hydrolysis method (i.e., the precipitation of an iron salt in basic media with a mild oxidant) (Sugimoto and Matijević, 1980). *In situ* polymer coating was achieved by adding PEI (25 kDa) during the reaction, as described previously (Calatayud et al., 2013). The particles exhibit a ferric oxide core (about 25 nm in diameter) and a thin polymer coating of PEI (about 0.7–0.9 nm); the total mass of PEI in the particles was around 14% (w/w) and the amount of the PEI on the particle surface was estimated 10 ± 2 μg/mg of NPs (Calatayud et al., 2013).

#### Preparation of NGF-PEI-NPs and NGF:PEI-NPs

NGF-β (Sigma, N1408) was marked with a fluorescent tag by labeling a protein mixture of NGF/BSA (1:6 w/w) with Alexa Fluor 488 (Life Technology, A-10235). TFP ester of the dye efficiently reacted with primary amines of proteins. Purification through a size exclusion resin allowed discarding the unincorporated dye; 90% of the initial protein was labeled with 1.5 moles of dye per mole of protein (*n* = 3). In order to functionalize the particles with the fluorescent NGF, 2 functionalization approaches were tested: covalent and non-covalent. PEI groups exposed on the surface of PEI-NPs offer primary and secondary amine-groups that have been used to functionalize the particles with the labeled protein.

In the covalent approach, the fluorescent NGF was attached to PEI-NP via EDAC chemistry (Fluka 03450, Sigma, USA). Briefly 0.6 mg EDAC and 1.2 mg NHS were dissolved in 0.2 ml 0.5 M MES-buffer (pH = 6.3) and added to 0.8 ml of protein (NGF concentration 35 μg/ml). After a few minutes, 500 μg of PEI-NPs were added and mixed for 3 h at 4–8°C. The unbounded protein was removed by magnetic separation and discharging the supernatant (3 washing steps). The NPs (thereafter labeled as NGF-PEI-NPs) were resuspended in PBS.

The non-covalent approach consisted in adding an equal volume of fluorescent protein (NGF concentration 35 μg/ml) to 1 mg/ml of PEI-NPs. The resulting suspension was dispersed at room temperature for 3 h under stirring. Unbound protein was removed by magnetic separation and discharging the supernatant (3 washing steps). The NPs (thereafter labeled as NGF:PEI-NPs) were resuspended in PBS.

For both approaches, the amount of fluorescent NGF bound to the surface of PEI-NPs was calculated by subtraction, i.e., by measuring the absorbance at 280 nm of the supernatant derived from the 3 washing steps. The concentration was calculated by using a calibration curve obtained with known amounts of fluorescent NGF. The composition of the sample was estimated to be 500 μg/ml of NPs, 7 μg/ml of NGF.

#### Validation of NGF:PEI-NPs respectively NGF-PEI-NPs

For validation of the functionality of the NGF bound to the PEI-NPs, PC12 cells were used as biological indicator. PC12 cells were incubated in serum-reduced media for 72 h either with NGF:PEI-NPs or NGF-PEI-NPs (NGF concentration 140 ng/ml, NP concentration 10 μg/ml) to induce differentiation of the cells based on functionalized PEI-NPs.

#### Preparation of GDNF:PEI-NPs

For NP functionalization, a protein mixture of GDNF/BSA (1:4 w/w, GDNF from Sigma Aldrich, USA) was labeled with Alexa Fluor 488. Purification through a size exclusion resin allowed discarding the unincorporated dye; 90% of the initial protein was labeled with 1.5 moles of dye per mole of protein (*n* = 3). The functionalization of the NPs was carried out by adding the labeled protein (concentration of GDNF 35 μg/ml) to PEI-NPs (500 μg/ml). The resulting suspension was dispersed at room temperature for 3 h under stirring. Unbound protein was discarded (via magnetic separation) and the functionalized NPs were resuspended in 15% glycerol water solution to stabilize the product. The composition of the sample was estimated to be 500 μg/ml of NPs, 2.8 μg/ml of GDNF, 11.2 μg/ml of BSA, 15% of glycerol.

#### Validation of GDNF:PEI-NPs

To validate the functionality of GDNF bound to PEI-NPs, we analyzed the survival of motor neurons of an organotypic spinal cord culture as biological indicator. Cultures were divided into 4 groups with 2 receiving GDNF supplemented to the media (100 ng/ml or 50 ng/ml GDNF), another without any GDNF supplement and 1 group cultivated with 10 μg/ml GDNF:PEI-NPs for the entire cultivation period of 1 week. Spinal cord slices of the GDNF:PEI-NPs group were pre-incubated for 30 min on ice with 20 μg/ml GDNF:PEI-NPs diluted in preparation buffer to increase NP-uptake. Slices of other groups were handled in parallel using preparation buffer without NPs.

To validate the stability of GDNF:PEI-NPs in serum-high media for spinal cord cultures, we pre-incubated 10 μg/ml GDNF:PEI-NPs in spinal cord media for 1 h at 4°C. Afterwards, GDNF:PEI-NPs were separated using a neodym magnet. One group received this media supernatant for cultivation (thereafter supernatant GDNF:PEI-NPs). The separated GDNF:PEI-NPs were resuspended in fresh, GDNF-free spinal cord media. This GDNF:PEI-NPs containing media was also used for cultivation (thereafter resuspended GDNF:PEI-NPs). Controls were fed with GDNF supplemented media (50 or 100 ng/ml) for the whole cultivation time and handled in parallel.

Statistical analysis was performed using a One-Way-ANOVA followed by a Bonferroni *post-hoc* analysis with *p* < 0.05 being statistical significant.

## Results

### Approaches for NGF functionalization: covalent and non-covalent

The fluorescently labeled NGF and the functionalized NPs were tested on PC12 cells as these possess specific cell surface receptors that bind NGF. In presence of this growth factor, cells undergo a dramatic change in phenotype wherein they acquire a large part of the characteristic properties of sympathetic neurons. PC12 cells treated with 50 ng/ml of fluorescent NGF were found to exhibit a phenotype similar to the cultures treated with 50 ng/ml of NGF. In a previous study Western blot analysis showed an upregulation of phosphorylated TrkA receptor expression by NGF-β, fluorescent labeled NGF, and fluorescently labeled NGF coupled PEI-NPs confirming this result (Riggio et al., 2014).

**Figure 1:**
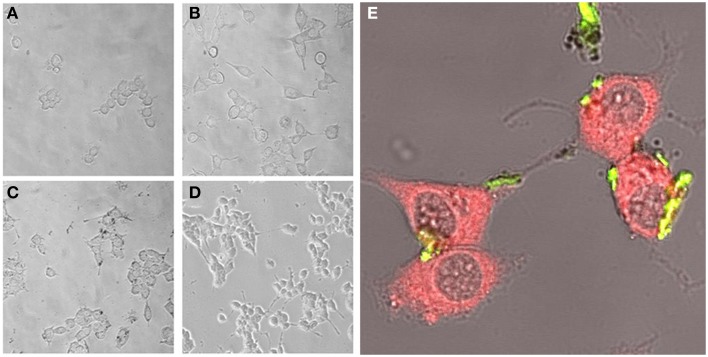
**PC12 cells incubated for 72 h with reduced media (A), reduced media modified with NGF (B), reduced media modified with NGF-PEI-NP (C), or reduced media modified with NGF:PEI-NP (D) (20X)**. Confocal microscopy of PC12 cells after 72 h of incubation with NGF:PEI-NPs is shown in **(E)**. Actin staining reveals the presence of NPs (green) in cell body, the growth cone at the tip of the axons of the differentiated cells (40X).

**Figure 2:**
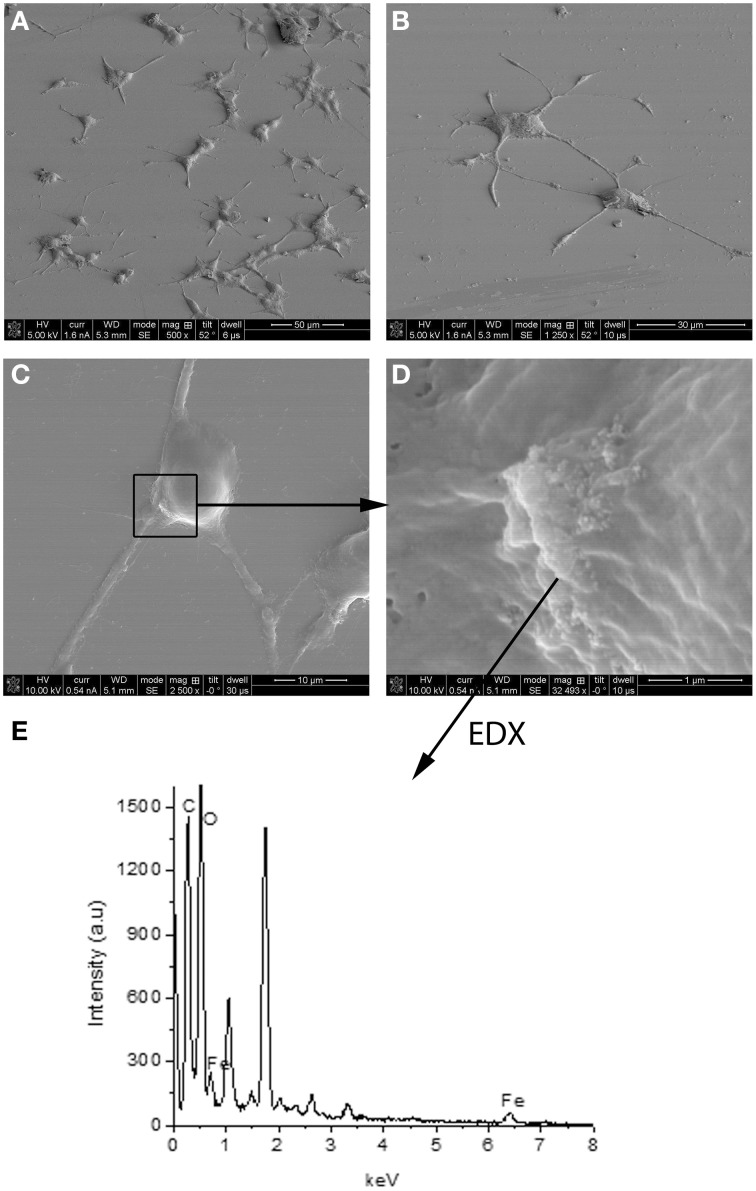
**SEM image of PC12 cells incubated 72 h with NGF:PEI-NPs (A–D)**. PC12 cells grew neurites confirming differentiation due to NGF **(A–C)**. Box in **(C)** points to NPs bound to the cell surface, **(D)** illustrates higher magnification of NPs and area of EDX analysis shown in **(E)**.

**Figure 3:**
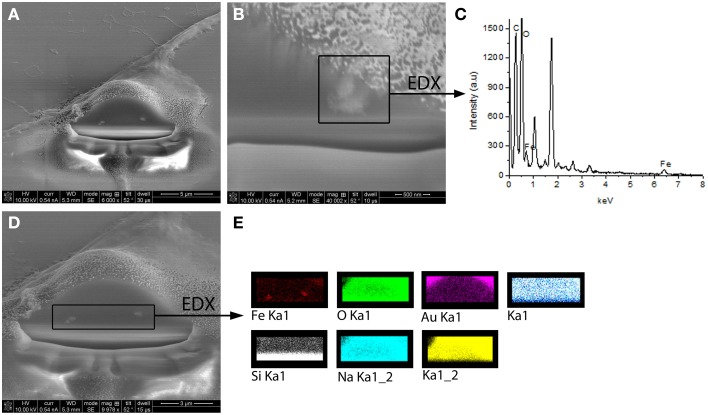
**FIB-SEM images of milled PC12 cells differentiated with NGF:PEI-NPs showing clear internalization of the NPs by PC12 cells (A,B,D)**. In **(B)** one cluster of internalized NPs was chosen for EDX analysis illustrated in **(C)**. EDX analysis revealed iron content of chosen cluster. **(D)** displays alternative EDX analysis area including both seen NPs clusters. In **(E)** EDX analysis of this area is shown and reveals iron content of the clusters confirming the internalization of iron oxide NPs.

Experimental results revealed that the fluorescent NGF binds the surface of the PEI-NPs at a ratio of 14 ± 3 μg of fluorescent NGF per mg of PEI-NPs. No statistical difference was found between non-covalent NGF:PEI-NPs and covalent NGF-PEI-NPs (data not shown). PC12 cells were incubated with reduced media modified with NGF:PEI-NPs or NGF-PEI-NPs (NGF concentration 140 ng/ml, NP concentration 10 μg/ml). In Figure 1, PC12 cultures incubated with reduced media (Figure 1A), reduced media and NGF (Figure 1B), reduced media and NGF-PEI-NPs (Figure 1C), and reduced media and NGF:PEI-NPs (Figure 1D) are shown. For NGF-PEI-MNPs, a strong reduction of the neurite number and length was observed compared to the control (NGF 140 ng/ml). Otherwise, for NGF:PEI-NPs cell differentiation proceeded similarly to the control cultures treated with NGF. Confocal imaging demonstrated that the NGF:PEI-NPs are strongly engulfed by cells, being localized in both the cell body and neurite protrusions (Figure 1E). Based on these results, we can conclude that the covalent approach impairs the bio-functionality of the protein. We postulate that the strong interaction between the particles surface and NGF induce the protein wrapping/adsorption onto the particle surface. This could strongly affect the protein mobility, ultimately altering its 3D structure and the interaction with the TrkA receptor. Based on these results, we considered the non-covalent approach to be the most promising. Electron microscopy confirmed that in presence of NGF:PEI-NPs, PC12 cells were properly differentiated. They exhibited long neurites, which were well connected within the network (Figures 2A,B). Figure 2C shows a representative cell with a cluster of NPs bound to the cell surface, which is highlighted in Figure 2D. EDX analysis revealed iron content and therefore confirmed the NGF:PEI-NP–cell interaction (Figure 2E). FIB-SEM analysis of milled PC12 cells, a technique that allows sectioning a cell and acquiring information on the constitutive elements inside (Riggio et al., 2014), illustrated the successful internalization of NGF:PEI-NPs (Figures 3A,B,D). EDX analysis, shown in Figures 3C,E, confirmed that internalized clusters seen in Figures 3A,B,D are from iron and therefore iron oxide PEI-NPs.

### Validation of bio-functionality of GNDF:PEI-Nps

**Figure 4:**
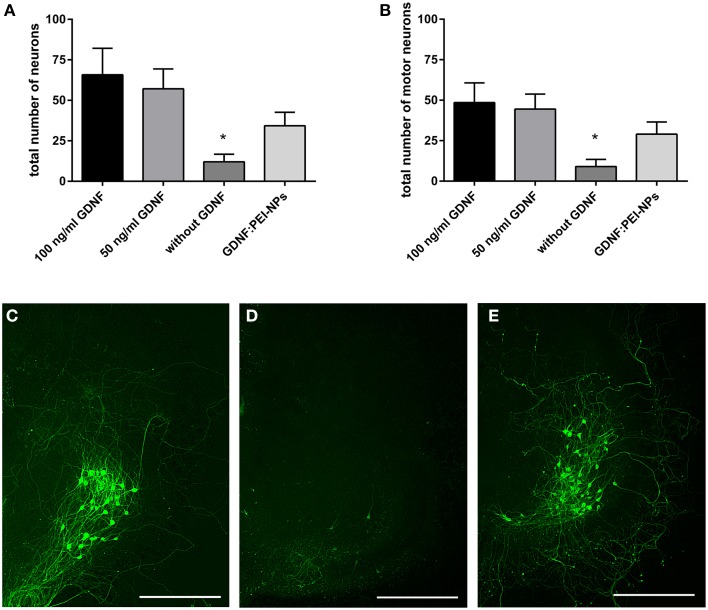
**Analysis of GDNF-functionality of GDNF:PEI-NPs**. Cultures were cultivated with GDNF supplemented to the media (100 or 50 ng/ml GDNF) as controls, without any GDNF supplement or with GDNF:PEI-NPs in the media. The total number of neurons (**A**, mean ± SEM) decreased in cultures without GDNF supplement, but there were no changes in the number of surviving neurons with incubation of GDNF:PEI-NPs compared to the controls. The same result was found concerning the number of surviving motor neuron (**B**, mean ± SEM). Corresponding fluorescent anti-pan-neurofilament staining of control culture **(C)**, culture cultivated without GDNF **(D)** and culture with GDNF:PEI-NPs **(E)**. Bars = 500 μm. Significance differences are marked with ^*^ and demonstrate *p* < 0.05.

First, we tested for bio-functionality of GDNF:PEI-NPs after coupling. For this, we analyzed the number of surviving motor neurons in organotypic spinal cord cultures incubated with these NPs instead of GDNF supplement via media. GDNF:PEI-NPs were able to keep motor neuronal populations alive. There were no significant differences in the total number of surviving neurons or in the number of motor neurons between controls containing 100 or 50 ng/ml GDNF in the media and GDNF:PEI-NPs incubated cultures (Figures 4A,B). Parallel handled cultures that were cultivated without GDNF supplement displayed significantly less surviving neurons in the total number and in the number of motor neurons than controls 100 (100 ng/ml GDNF supplement). Figures 4C–E show corresponding immunofluorescent stainings for anti-pan-neurofilament. Controls and cultures, incubated with GDNF:PEI-NPs illustrated a prominent motor neuronal population (Figures 4C,E). Cultures without GDNF supplement displayed a clear reduction of stained motor neurons (Figure 4D). Accordingly, GDNF function was kept during coupling to the PEI-NPs.

The non-covalent approach was used to test the NP functionalization with GDNF. Therefore, we checked the GDNF coupling stability to the PEI-NPs. For this, organotypic cultures were cultivated for 7 days and fed with media containing 100 ng/ml GDNF (control 100), 50 ng/ml GDNF (control 50, reduced GDNF to compare with the lower GDNF concentration, which is delivered by the GDNF:PEI-NPs), supernatant of GDNF:PEI-NPs or resuspended GDNF:PEI-NPs.

GDNF:PEI-NPs were diluted in spinal cord media without supplemented GDNF (NP concentration 10 μg/ml, GDNF concentration about 55 ng/ml) and incubated for 1 h at 4°C. Then, NPs were separated from media with a magnet and the NPs were resuspended in fresh media without GDNF and used for cultivation of the resuspended GDNF:PEI-NPs-group. The supernatant, which was left after the magnetic separation, was also used for cultivation (supernatant GDNF:PEI-NPs). GDNF is required for the survival of the motor neuronal population in the spinal cord cultures. Therefore, we quantified the number of surviving motor neurons in both groups and compared them to controls. If the number of motor neurons of the resuspended GDNF:PEI-NPs-group was similar to the number of motor neurons in the controls, then the GDNF was delivered by the GDNF:PEI-NPs. But if the number of motor neurons of the supernatant group were similar to that of the controls then the GDNF was dissociated from the PEI-NPs and left in the supernatant.

**Figure 5:**
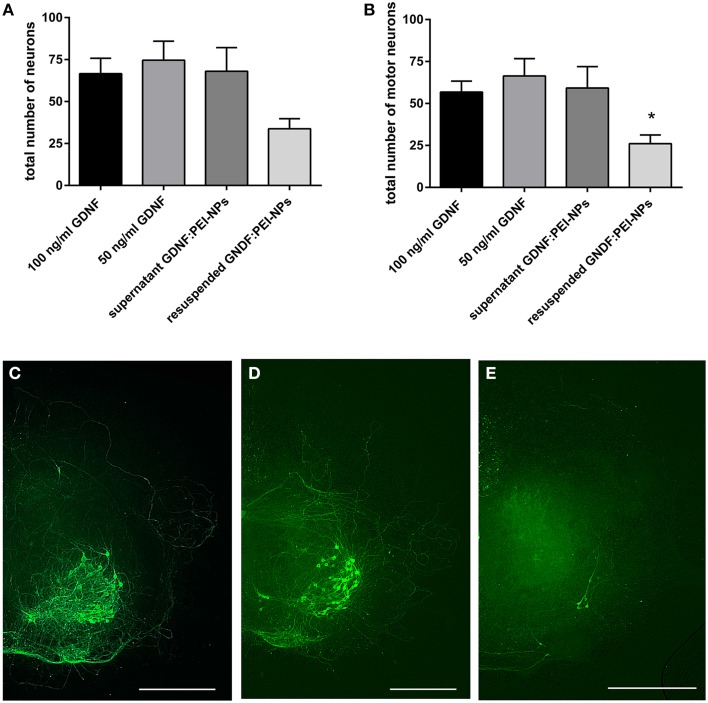
**Analysis of coupling stability of GDNF to PEI-NPs in spinal cord cultures**. Cultures were cultivated with GDNF supplemented to the media (100 or 50 ng/ml GDNF) as controls, with the supernatant of previous suspended GDNF:PEI-NPs (supernatant GDNF:PEI-NPs) or with resuspended GDNF:PEI-NPs in fresh media. The total number of neurons (**A**, mean ± SEM) did not show significant differences, but the number of motor neurons **(B)** was significantly decreased in cultures cultivated with resuspended GDNF:PEI-NPs compared to control (50 ng/ml GDNF). Corresponding fluorescent anti-pan-neurofilament stainings of control culture **(C)**, culture cultivated with supernatant of GDNF:PEI-NPs **(D)**, and culture cultivated with GDNF:PEI-NPs **(E)**. Bars = 500 μm. Significance differences are marked with ^*^ and demonstrate *p* < 0.05.

Figure 5 displays the result. Organotypic cultures incubated with the supernatant of former diluted GDNF:PEI-NPs revealed the same survival rate of the total number of neurons and the motor neurons as was seen in the controls (Figures 5A,B). At the same time, cultures that were cultivated with the resuspended GDNF:PEI-NPs showed a significant decrease of the number of surviving motor neurons compared to control 50. Figures 5C–E shows corresponding immunofluorescent stainings for anti-pan-neurofilament. Controls and cultures incubated with the supernatant of GDNF:PEI-NPs, but not with the particles itself, illustrated a pronounced motor neuronal population (Figures 5C,E). Cultures receiving resuspended GDNF:PEI-NPs showed a clear reduction of stained motor neurons (Figure 5D). So, GDNF bio-functionality did not correspond to the availability of the GDNF:PEI-NPs. The GDNF coupling was not stable.

## Discussion

### Functionalization with NGF

Aim of this study was to functionalize magnetic NPs with growth factors to facilitate a receptor-mediated intracellular uptake into neurons. Enhanced neuronal uptake would allow the magnetically targeted delivery of drugs and growth factors to injury sites of the CNS and PNS.

There are only a few studies using growth factors to functionalize NPs. Polak and Shefi (2015) published an overview about growth factors used. Besides NGF, basic fibroblast growth factor (FGF2), BDNF, and GDNF are reported.

To check the bio-functionality of growth factors after binding to NPs a fast, easy, and reliable model is needed. *In vitro* assays are the methods of choice because they require no or fewer animals, are faster, and also cheaper than *in vivo* models.

In our study, we first chose NGF for loading the self-made magnetic PEI-NPs. For determining the bio-functionality of the NGF loaded PEI-NPs, we used PC12 cells as indicator model. PC12 cells can be differentiated with NGF into cells displaying functions of sympathetic neurons. NGF-treated cells terminate proliferation, develop neurites, become electrically excitable, and express neuronal proteins like synapsin and growth associated protein 43 (GAP43) (Greene and Tischler, 1976; Fujita et al., 1989; Das et al., 2004). The outgrowth of neurites is used as a differentiation marker because it is easy to quantify. PC12 cells are used in many studies concerning NPs and functionalization of NPs, e.g., Pisanic et al. (2007) used PC12 cells to analyze nanotoxicity of iron oxide NPs, Roy et al. (2010) used PC12 cells to check for the targeting of NGF-conjugated NPs and evaluated the expression levels of Trk- and p75-receptors, and Mittnacht et al. (2010) functionalized siRNA for RhoA, a kinase playing a role in neurite outgrowth. We started with a covalent approach to bind NGF to the amine groups of PEI-NPs via EDAC chemistry. Covalent binding worked, but obviously changed the structure of NGF, as these NGF-PEI-NPs were not able to differentiate PC12 cells properly. It seems that the protein function was impaired by covalent binding to PEI-NPs. NGF binds to its receptor TrkA via N-terminal residues. The loops L2 and L4 of the protein structure appear to be responsible for bio-functionality of NGF (Wiesmann et al., 1999). The covalent binding of NGF to amine groups of the PEI-NPs occurs randomly. Because we observed an impairment of bio-functionality of NGF, it is possible that binding to N-terminal residues or in the loops L2 and L4 took place. Alternatively, we bound NGF using a non-covalent approach, which is based on electrostatic interactions between molecules. These NGF:PEI-NPs were able to differentiate PC12 cells to neuronal cells. Functionality was maintained during binding to the PEI-NPs. Interestingly, experimental data demonstrated that the NGF:PEI-NPs complex is also stable when mixed to the cell culture media, which indicates that non-specific adsorption of plasma proteins does not alter the stability and the integrity of the conjugate (Riggio et al., 2013).

### Functionalization with GDNF

Additionally, we used GDNF for functionalization of our PEI-NPs. GDNF was first described in 1993 by Lin et al. (1993) as a growth promoting factor for embryonic midbrain dopaminergic neurons. Since then, it was shown that GDNF also promotes the survival of dopaminergic neurons in rodents and non-human Parkinson models (Gash et al., 1998), that it can prevent the programmed cell death of motor neurons during embryonic development (Oppenheim et al., 1995), and that GDNF increases survival and neurite sprouting in models of spinal cord injury (Li et al., 1995; Pajenda et al., 2014). GDNF also has non-neurotrophic functions: it regulates the ureteric branching during embryogenesis (Vega et al., 1996) and spermatogenesis (Meng et al., 2000). The various functions of GDNF limit the choice of useful *in vitro* models. One model that allows analyzing the GDNF functionality fast and reliably is the organotypic culture of dorsal root ganglions. The group of Ziv-Polat et al. (2014) measured sprouting and the onset of myelination to evaluate GDNF function. In contrast, we used a neonatal organotypic spinal cord culture to assess the bio-functionality of GDNF. In such cultures, GDNF is known to be a prominent neurotrophic factor for motor neuronal survival, as it is able to keep about 60% of the motor neurons alive during cultivation (Rakowicz et al., 2002; Vyas et al., 2010). Therefore, quantifying the number of surviving motor neurons after a sufficient cultivation time already allows one to judge the successful functionalization of NPs.

Based on our previous results of NGF coupling to PEI-NPs, we decided to use a non-covalent approach to bind GDNF to our PEI-NPs. To check the functionality of the bound GDNF, we cultivated our organotypic spinal cord cultures with GDNF:PEI-NPs only and compared the number of surviving motor neurons and the total number of neurons after 7 days of cultivation. Compared to control cultures, which received GDNF via media, cultures incubated with GDNF:PEI-NPs showed a similar total number of surviving neurons and also motor neurons at the end of the cultivation. Therefore, GDNF function was kept during coupling to PEI-NPs. Because of the 3-dimensional structure of the organotypic culture, visualizing an uptake of GDNF:PEI-NPs into neurons is difficult. Moreover the use of serum-high media for the cultivation of organotypic spinal cord cultures can cause problems because the NPs become surrounded by a protein corona (Lundqvist et al., 2008). Thus, we tried to confirm the stability of the non-covalent binding of GDNF to the PEI-NPs. According to the protocol already used to test the stability of NGF:PEI-NPs in biological media (Riggio et al., 2013), we incubated the GDNF:PEI-NPs in the serum-rich media for these cultures. After separating these NPs with a strong magnet, we used either the supernatant of the NPs for cultivation of the spinal cord cultures or resuspended them again in GDNF-free, fresh media and used this for the cultivation. If now the number of surviving motor neurons in cultures incubated with resuspended GDNF:PEI-NPs was similar to the number of surviving motor neurons in controls (receiving GDNF via media), the GDNF functionality was kept by the GDNF:PEI-NPs. If the number of surviving motor neurons in cultures cultivated with the supernatant (no GDNF:PEI-NPs left) was similar to the number of surviving motor neurons of the controls, the GDNF bio-functionality passed on to the media. In our study, the number of surviving motor neurons was not significantly different between controls and cultures incubated with the supernatant. But it decreased significantly in cultures cultivated with resuspended GDNF:PEI-NPs compared to controls. Therefore, the GDNF bio-functionality was kept in the supernatant and the GDNF separated from the PEI-NPs.

In contrast, non-covalently NGF:PEI-NPs were found to be stable in binding. They were up taken into PC12 cells and induced a differentiation of PC12 cells. One main difference between the media of PC12 cells and organotypic spinal cord cultures is the amount of serum. Spinal cord culture media contains 25% FBS compared to the 2% used in the media for differentiation of PC12 cells. As already mentioned, NPs are coated with a protein corona in serum-containing media (Lundqvist et al., 2008; Calatayud et al., 2014). The protein corona contains a variety of proteins like immunoglobulins, albumin, apolipoproteins, fibrinogen, and many more (Lundqvist et al., 2008). Non-covalent coupling of GDNF to PEI-NPs is not a fixed coupling; it is based on electrostatic interactions between residues of molecules. These interactions are weaker than covalent interactions where atoms share electrons. Due to the high serum content in the spinal cord media, it can be assumed that the protein corona is more pronounced than in the low-serum media of the PC12 cells. Thus, serum proteins and GDNF appear to compete for binding, which results in a loss of GDNF bound to PEI-NPs.

The group of Ziv-Polat et al. (2014) compared the bio-functionality of NGF, FGF2, and GDNF conjugated to iron oxide NPs. They showed that the conjugated neurotrophic factors were more stable and also the bio-functionality of the growth factors increased significantly. In particular, GDNF bound to the NPs enhanced the myelination in their organotypic dorsal root ganglion model. To conjugate all 3 neurotrophic factors, Ziv-Polat et al. (2014) used a covalent approach via activated double bonds of dextran- or gelatin-coated NPs. Also Marcus et al. (2015) used a covalent binding strategy to bind NGF to human serum albumin-coated iron oxide NPs. They successfully induced the differentiation of PC12 cells in a neuronal-like cell type, thereby proving the bio-functionality of NGF-HSA-NPs. In our experiments, covalently binding was shown to impair the bio-functionality of NGF. We used PEI-coated iron oxide NPs, which allow binding covalently proteins via amine groups. Obviously, binding proteins covalently to NPs is dependent on the chosen chemical strategy. Interactions between proteins and NPs are hardly predictable and since the exact binding site in the structure of the protein is somehow random, the outcome regarding the bio-functionality seems to be random as well. Even if the catalytic site is not involved in chemical binding, the overall 3D structure of the protein could be affected by the strong chemical bonding with the particle, resulting in structural deformation, and loss of function. Designed, small peptides could help to solve these issues and prevent unintentional binding effects and loss of functionality. Alternative approaches already tried to develop small peptides mimicking growth factor functions. Smaller peptides have certain advantages in comparison to native proteins. Growth factors address various signal pathways leading to a variation of actions which can include unintentional effects, too (Boyd and Gordon, 2003). GDNF overexpression during embryogenesis, for example, is preventing naturally occurring programmed cell death of motor neurons leading to hyperinnervation of muscles (Zwick et al., 2001). Small peptides which are designed with special functional sites give the chance to generate distinct signaling profiles. Forte et al. (2014) described the binding of 2 growth factor mimicking peptides, NGF (1–14) and BDNF (1–12) on a gold carrier, a chemistry which can be used to conjugate to, e.g., gold nanoparticles. Previously, the same group reported biological actions of a NGF (1–14) peptide which could activate single intracellular signal cascades in PC12 cells (Travaglia et al., 2015). Bradley et al. (2010) specified another synthetic peptide showing GDNF functions. This peptide DNSP-11from the proGDNF-domain was shown to support neuronal survival and neuritic outgrowth *in vitro*, but also increasing dopamine levels in an animal model of Parkinson disease.

Taken together, we could show that functionalization of NPs with growth factors can be complex and difficult. Functionalization of PEI-NPs with NGF using covalent and non-covalent binding strategies displayed contrasting results. Covalently bound NGF-PEI-NPs were not able to differentiate PC12 cells, proving a lack of bio-functionality after binding. Non-covalent bound NGF:PEI-NPs differentiated PC12 cells into neuronal-like cells, thereby showing bio-functionality of NGF. Due to the experiences of NGF-functionalization, we used a non-covalent approach for functionalization of PEI-NPs with GDNF. This was shown to keep bio-functionality of GDNF in an organotypic spinal cord model, but was additionally found to be unstable bound to the PEI-NPs. We suspect that the different serum content in the media of PC12 cells and organotypic spinal cord cultures is a problem for using non-covalent approaches. All in all, our study shows the importance of checking the bio-functionality of growth factors bound to NPs with a proper biological model. The final future aim of all studies using NPs in life sciences is to use these particles in humans. For this, it is important to develop reproducible models that allow a prognosis of NPs action *in vivo*.

### Conflict of interest statement

The authors declare that the research was conducted in the absence of any commercial or financial relationships that could be construed as a potential conflict of interest.

## Abbreviations

BSA: bovine serum albumin
BDNF: brain-derived growth factor
CNS: central nervous system
FBS: fetal bovine serum
GFR-α: GDNF family receptor-α
GDNF: glial cell-line derived neurotrophic factor
MRI, magnetic resonance imaging, MAPK: mitogen-activated protein kinase
NPs: nanoparticles
NGF: nerve growth factor
PNS: peripheral nervous system
PBS: phosphate buffered saline
PI3K: phosphatidylinositol 3-kinase
PLCγ: phospholipase Cγ
PEI: polyethyleneimine
PC12: rat pheochromocytoma cell line
TrkA: tropomyosin receptor kinase A.
